# Supplementary material for: Monitoring of Serum Potassium and Calcium Levels in End-Stage Renal Disease Patients by ECG Depolarization Morphology Analysis
Source: Sensors (Basel). 2022 Apr 12;22(8):2951. doi: 10.3390/s22082951 (PMC9027214; doi:10.3390/s22082951)
Supplement: Supplementary file 1 [file sensors-22-02951-s001.zip › sensors-1635619-supplementary-update.pdf]

Article

Monitoring of Serum Potassium and Calcium Levels in End-stage Renal Disease Patients by ECG Depolarization Morphology Analysis

Hassaan A. Bukhari [1,2,3,4,*], Carlos Sánchez [1,2], José Esteban Ruiz [5], Mark Potse [3,4], Pablo Laguna [1,2] and Esther Pueyo [1,2]

1 BSICoS Group, I3A Institute, IIS Aragón, University of Zaragoza, 50018 Zaragoza, Spain; cstapia@unizar.es (C.S.); laguna@unizar.es (P.L.); epueyo@unizar.es (E.P.)
2 CIBER en Bioingeniería, Biomateriales y Nanomedicina (CIBER-BBN), 50018 Zaragoza, Spain
3 Carmen Team, Inria Bordeaux—Sud-Ouest, 33405 Talence, France; mark@potse.nl
4 Université de Bordeaux, IMB, UMR 5251, 33400 Talence, France;
5 Nephrology Department, Hospital Clínico Universitario Lozano Blesa, 50009 Zaragoza, Spain; jeruizl@salud.aragon.es
* Correspondence: hassaanahmed01@unizar.es

Citation: Bukhari, H.A.; Sánchez. C.; Ruiz, J.E.; Potse, M.; Laguna, P.; Pueyo, E. Monitoring of Serum Potassium and Calcium Levels in End-Stage Renal Disease Patients by ECG Depolarization Morphology Analysis. Sensors **2022**, *22*, 2951. https://doi.org/10.3390/s22082951

Academic Editors: Riccardo Pernice, Massimo W. Rivolta and Raquel Bailón

Received: 2 March 2022
Accepted: 7 April 2022
Published: 12 April 2022

Publisher's Note: MDPI stays neutral with regard to jurisdictional claims in published maps and institutional affiliations.

Copyright: © 2022 by the authors. Licensee MDPI, Basel, Switzerland. This article is an open access article distributed under the terms and conditions of the Creative Commons Attribution (CC BY) license (https://creativecommons.org/licenses/by/ 4.0/).

Figure S1 represents the QRS complexes in an ECG segment of a particular patient. The QRS complexes with non-dominant polarity are plotted in magenta and the QRS complexes with the dominant polarity are shown in black.

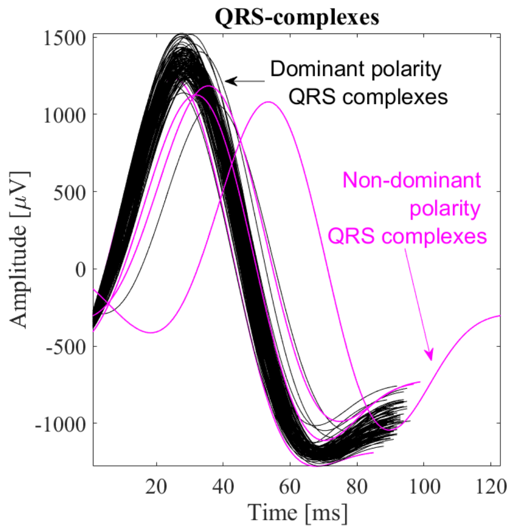

Figure S1. QRS complexes, including selected dominant QRS complexes (black) and non-dominant polarity QRS complexes (magenta) from a particular patient at a particular time.

Table S1 shows the Pearson correlation coefficient to assess the poor association between QRS- ($d^{u}_{w,Q}$) and T wave- ($d^{u}_{w,T}$) based multivariable estimators, using stage-specific, S, patient-specific, P, and global, G, approaches, thus implying that using both markers together for $[K^{+}]$ and $[Ca^{2+}]$ estimation could improve the results.

Figure S2 shows the relative errors e_r for all patients and HD stages in the estimation of $[K^{+}]$ and $[Ca^{2+}]$ using stage-specific (top panel), patient-specific (middle panel) and global (bottom panel) approaches.

Bland-Altman plots in Figures S3-S8 show the difference vs the mean of actual and estimated $[K^{+}]$ and $[Ca^{2+}]$ for $d^{u}_{w,Q}$, $d^{u}_{w,T}$ and their combination using stage-specific, patient-specific and global approaches.

In addition to that, Bland-Altman plots in Figures S9-S12 show the difference vs the mean of actual and estimated $[K^{+}]$ and $[Ca^{2+}]$ for previously proposed T wave markers

Table S1. Pearson correlation coefficient (r) to assess the association between QRS ($d^{u}_{w,Q}$) and T waves ($d^{u}_{w,T}$) based multivariable estimators, using stage-specific, S, patient-specific, P, and global, G, approaches.

$d^{u}_{w,Q}$ vs. $d^{u}_{w,T}$	S	P	G
h_0	0.23		
h_1	0.06		
h_2	−0.04		
h_3	0.21		
h_{4-}	0.05		
h_{48}	−0.17		
Median		0.75	
IQR		0.29	
Global			0.28

from other authors $T_{S/A}$ (Corsi et al., 2012), and $T_{S/\sqrt{A}}$ (Attia et al., 2016), using stage-specific, patient-specific and global approach. Multivariable estimators combining information from $d^{u}_{w,Q}$ and $d^{u}_{w,T}$ outperformed univariable estimators (the ones proposed by us and other authors).

Note: Here, we showed the comparison with previously proposed T wave markers only because no previous studies investigated changes in the whole morphology of QRS complex at varying $[K^+]$ and $[Ca^{2+}]$.

As can be seen from the Table S2, the association between the QRS width and the RR interval was remarkably higher for some of the patients (highly positive in Patients 1–10, highly negative in Patients 11–16), it showed moderate (Patients 17–22) or poor (Patients 23–29) correlation for other patients. Therefore, the median value of r between QRS_w and RR is relatively low, 0.42 in all the 29 ESRD patients.

Table S2. Pearson correlation coefficient (r) between QRS complex width and RR interval in 29 ESRD patients. Values are expressed as median (interquartile range) over patients.

QRS_w	P1–10	P11–16	P17–P22	P23–P29	P1–P29 (overall)
r	0.77 (0.17)	−0.73 (0.21)	0.45 (0.13)	−0.04 (0.35)	0.42 (0.91)

Figure S13 shows the variations in sodium ($[Na^+]$) during HD in our analyzed ESRD patients. Table S3 shows the p-values between each consecutive HD point and we did not find significant differences, the one we found for $[K^+]$ and $[Ca^{2+}]$ (Figure 4, panels g-h, of main manuscript). Table S4 shows Pearson (r) and Spearman (ρ) correlation coefficient between all the analyzed QRS complex markers and $[Na^+]$.

Table S3. P-values from the Wilcoxon signed-rank test to assess differences in $[Na^+]$ between consecutive time stages.

p-value	h_0–h_1	h_1–h_2	h_2–h_3	h_3–h_4
$[Na^+]$	0.17	0.06	0.39	0.16

As can be seen from the Table S5, the association between the QRS width and the $[Na^+]$ was remarkably high for some of the patients (highly positive in Patients 1–12, highly negative in Patients 13–14), whereas it showed moderate (Patients 15–18) or poor (Patients

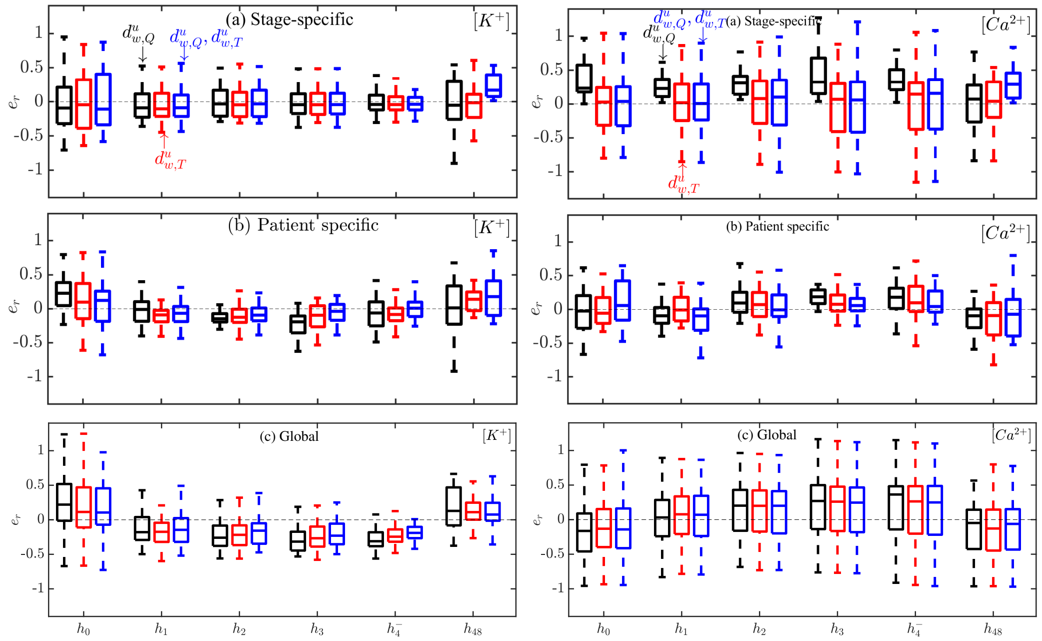

Figure S2. Box plots of $[K^+]$ and $[Ca^{2+}]$ estimation errors e_r along HD stages for all patients using $d^u_{w,Q}$(black), $d^u_{w,T}$(red) and the combination of $d^u_{w,Q}$ and $d^u_{w,T}$ (blue) for stage-specific (top), patient-specific (middle) and global (bottom) approaches. The central line indicates the median, whereas top and bottom edges show the 25-th and 75-th percentiles.

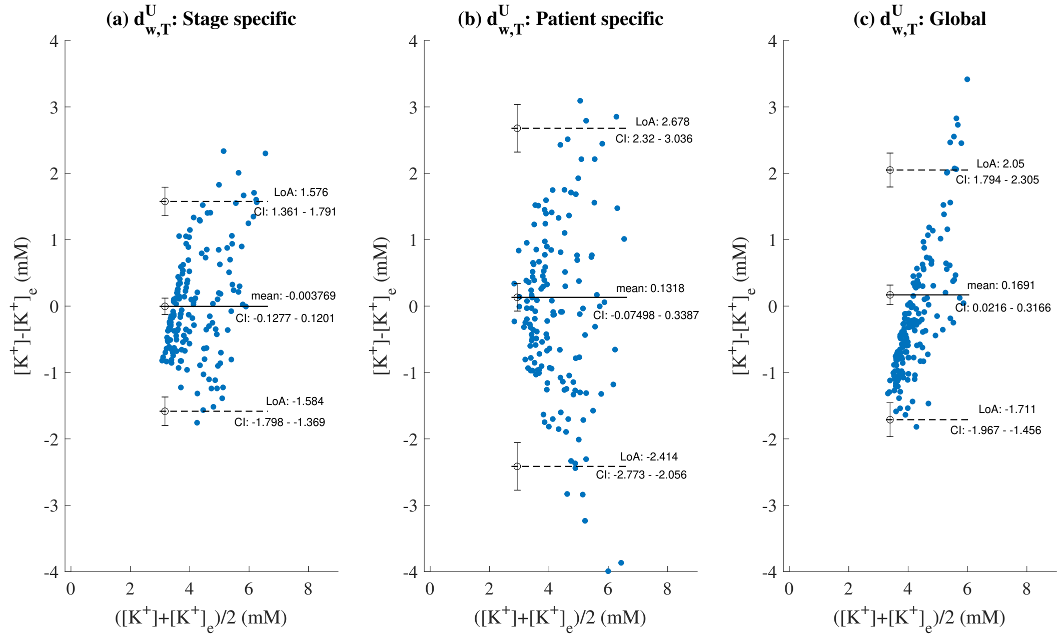

Figure S3. Left Panel: Bland-Altman plot between actual and estimated $[K^+]$ for $d^u_{w,T}$ using stage-specific approach. Middle Panel: Bland-Altman plot using patient-specific approach. Right Panel: Bland-Altman plot using global approach.

19–29) correlation for other patients. This caused the median value of r between QRS_w and $[Na^+]$ to be relatively low (0.24) in the cohort of 29 ESRD patients.

Tables S6–S7 show a comparison between mean absolute errors obtained for the analyzed markers analyzed in this study and in previous studies.

Tables S8–S9 show a comparison between root mean square errors obtained for the analyzed markers analyzed in this study and in previous studies.

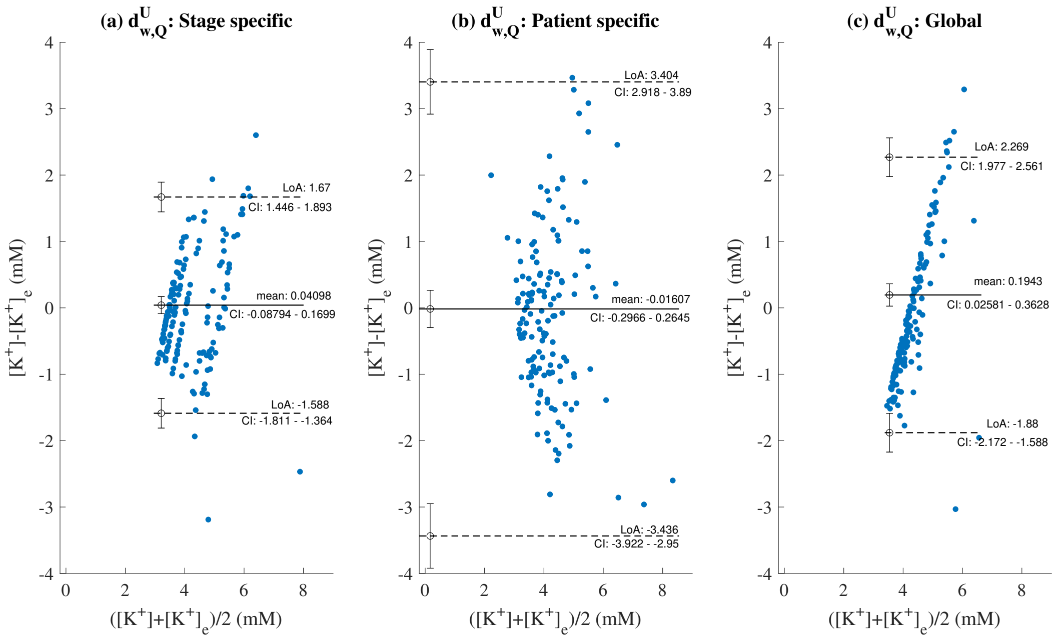

Figure S4. Left Panel: Bland-Altman plot between actual and estimated $[K^+]$ for $d^{u}_{w,Q}$ using stage-specific approach. Middle Panel: Bland-Altman plot using patient-specific approach. Right Panel: Bland-Altman plot using global approach.

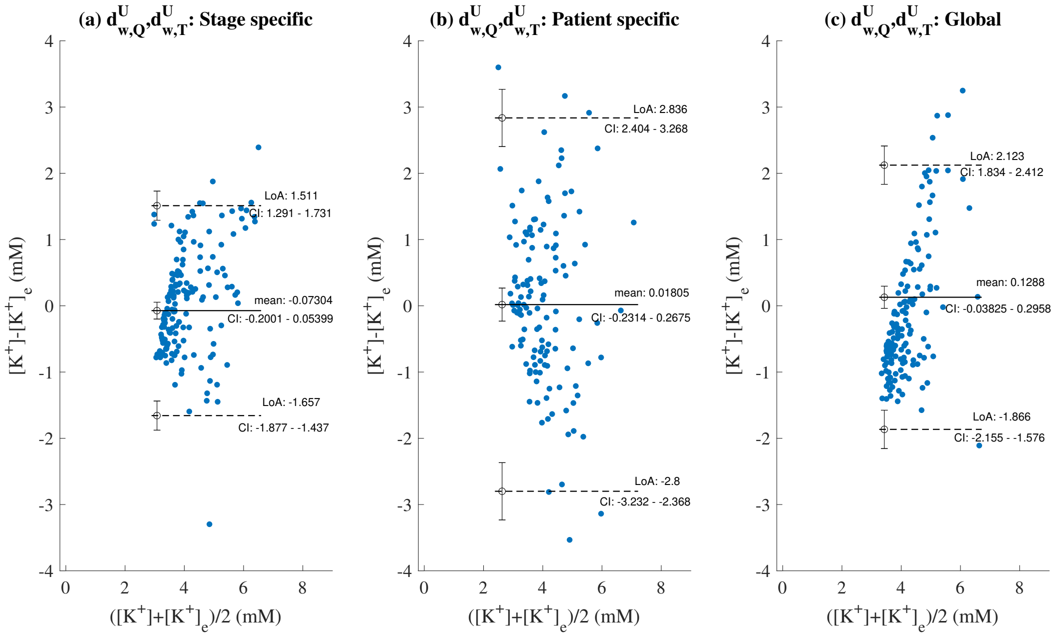

Figure S5. Left Panel: Bland-Altman plot between actual and estimated $[K^+]$ for the combined $d^{u}_{w,Q}$ and $d^{u}_{w,T}$ using stage-specific approach. Middle Panel: Bland-Altman plot using patient-specific approach. Right Panel: Bland-Altman plot using global approach.

Table S4. Pearson (r) and Spearman (ρ) correlation coefficients between QRS complex markers and $[Na^+]$ in 29 ESRD patients along HD. Values are expressed as median (interquartile range) over patients.

	QRS_w	QRS_a	$d^{u}_{w,Q}$	$d_{a,Q}$	$d^{NL}_{w,Q}$	$d^{NL}_{a,Q}$
r	0.24 (1.01)	0.30 (0.97)	−0.31 (1.00)	−0.37 (1.18)	−0.33 (1.05)	−0.18 (1.13)
ρ	0.23 (0.84)	0.24 (0.99)	0.00 (0.98)	−0.29 (0.90)	−0.10 (0.96)	−0.11 (0.95)

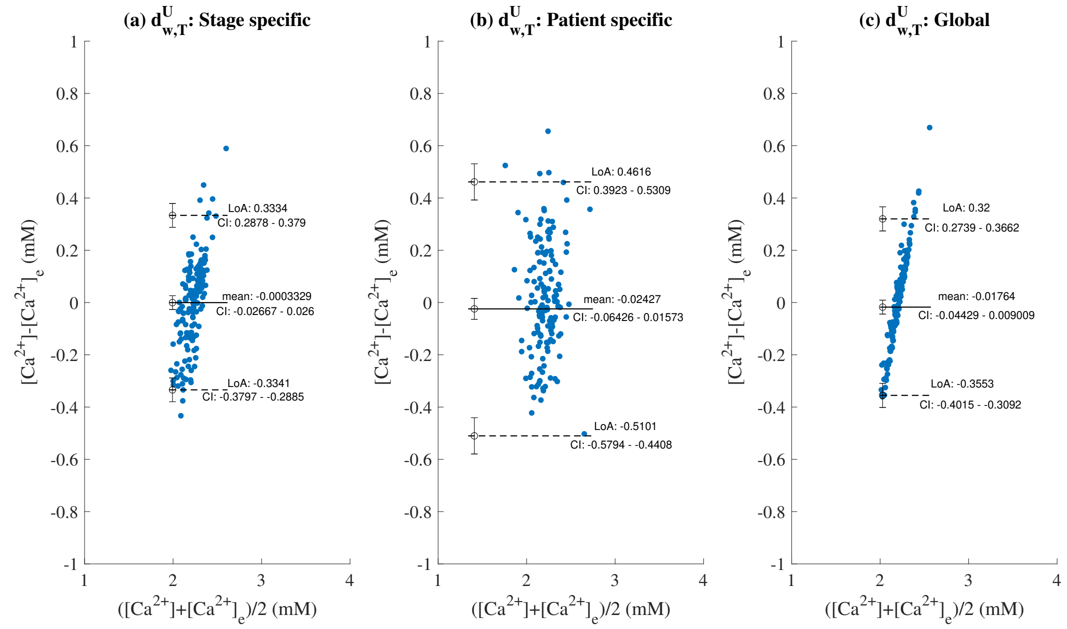

Figure S6. Left Panel: Bland-Altman plot between actual and estimated $[Ca^{2+}]$ for $d^{u}_{w,T}$ using stage-specific approach. Middle Panel: Bland-Altman plot using patient-specific approach. Right Panel: Bland-Altman plot using global approach.

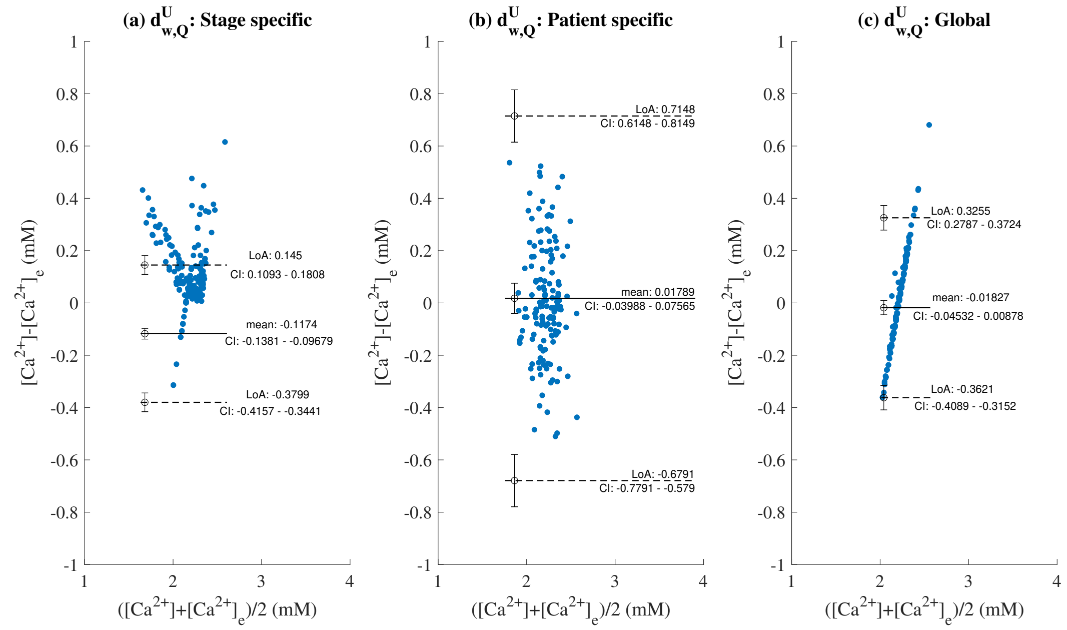

Figure S7. Left Panel: Bland-Altman plot between actual and estimated $[Ca^{2+}]$ for $d^{u}_{w,Q}$ using stage-specific approach. Middle Panel: Bland-Altman plot using patient-specific approach. Right Panel: Bland-Altman plot using global approach.

Table S5. Pearson correlation coefficient (r) between QRS complex width and $[Na^{+}]$ in 29 ESRD patients. Values are expressed as median (interquartile range) over patients.

QRS_w	P1–12	P13–14	P15–P18	P19–P29	P1–P29 (overall)
r	0.88 (0.13)	−0.72 (0.05)	−0.40 (0.23)	−0.05 (0.22)	0.24 (1.01)

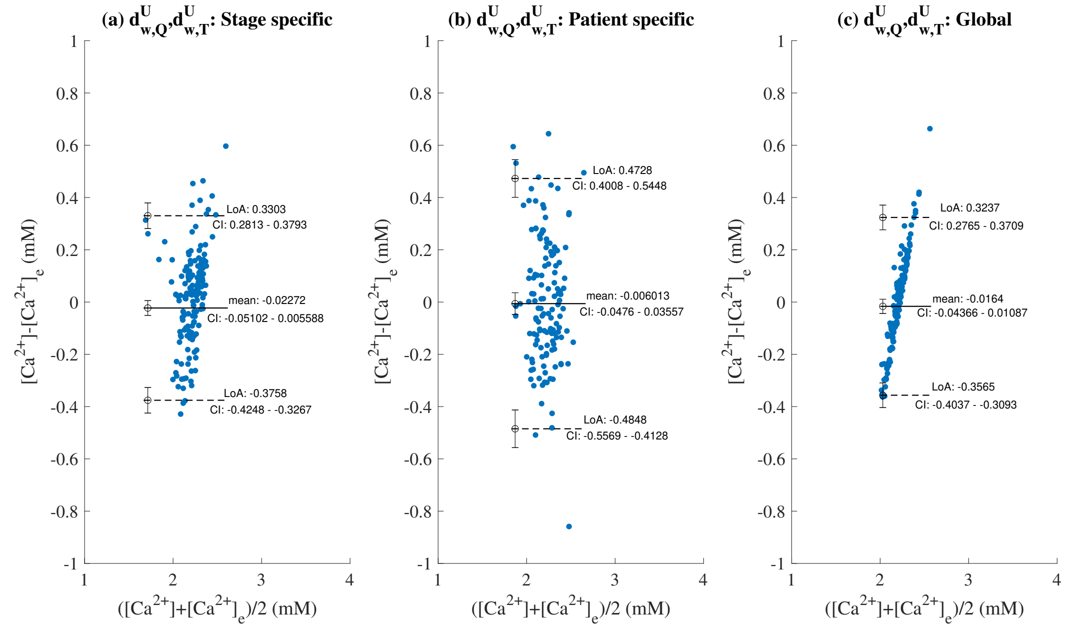

Figure S8. Left Panel: Bland-Altman plot between actual and estimated $[Ca^{2+}]$ for the combined $d^u_{w,Q}$ and $d^u_{w,T}$ using stage-specific approach. Middle Panel: Bland-Altman plot using patient-specific approach. Right Panel: Bland-Altman plot using global approach.

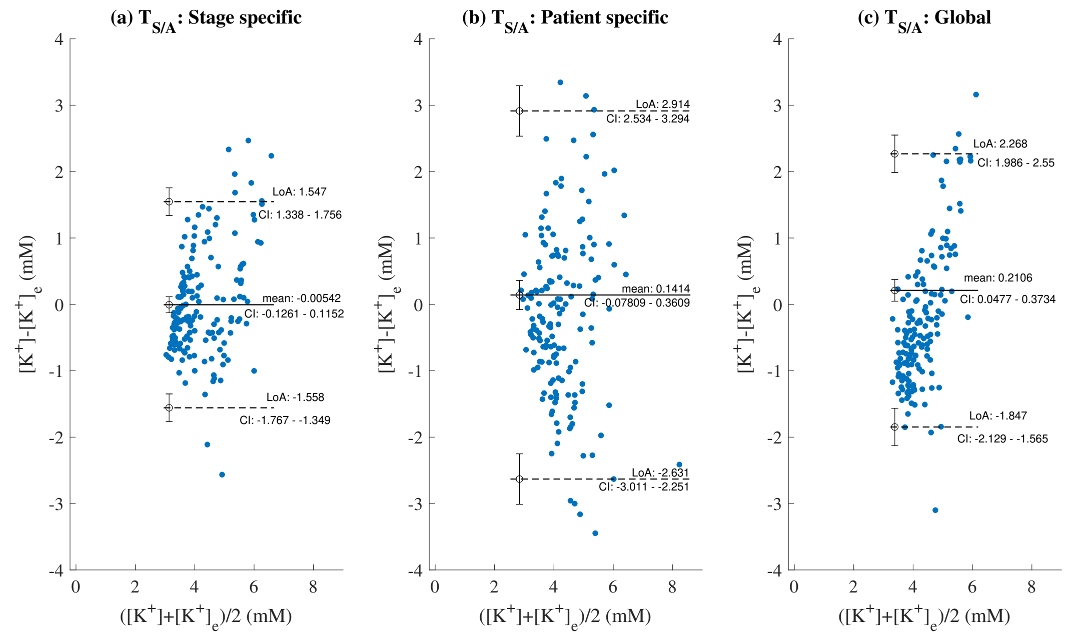

Figure S9. Left Panel: Bland-Altman plot between actual and estimated $[K^+]$ for $T_{S/A}$ using stage-specific approach. Middle Panel: Bland-Altman plot using patient-specific approach. Right Panel: Bland-Altman plot using global approach.

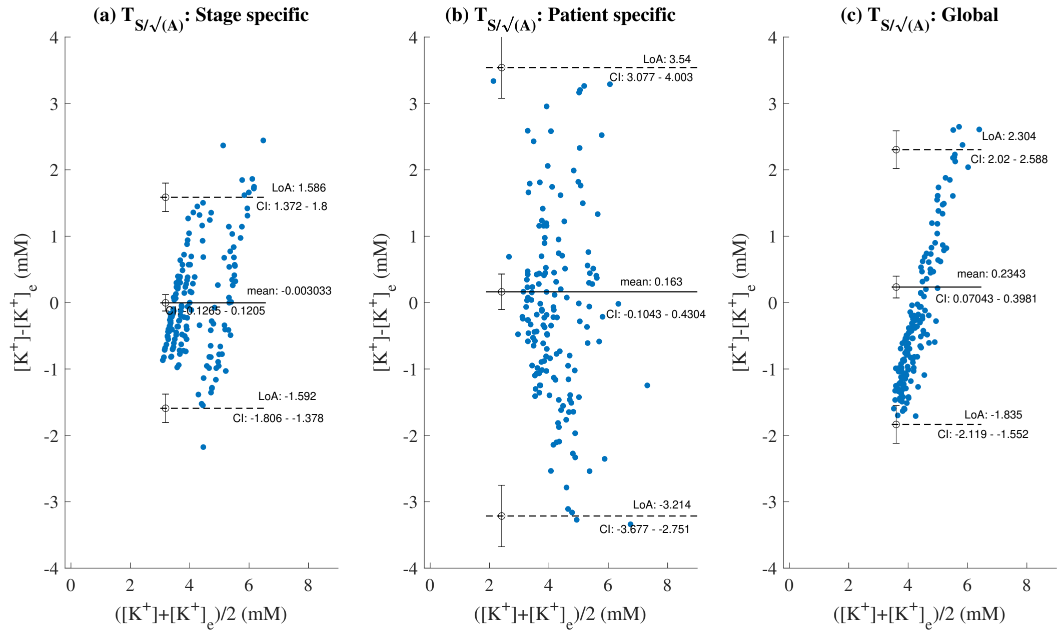

Figure S10. Left Panel: Bland-Altman plot between actual and estimated $[K^+]$ for $T_{S/\sqrt{A}}$ using stage-specific approach. Middle Panel: Bland-Altman plot using patient-specific approach. Right Panel: Bland-Altman plot using global approach.

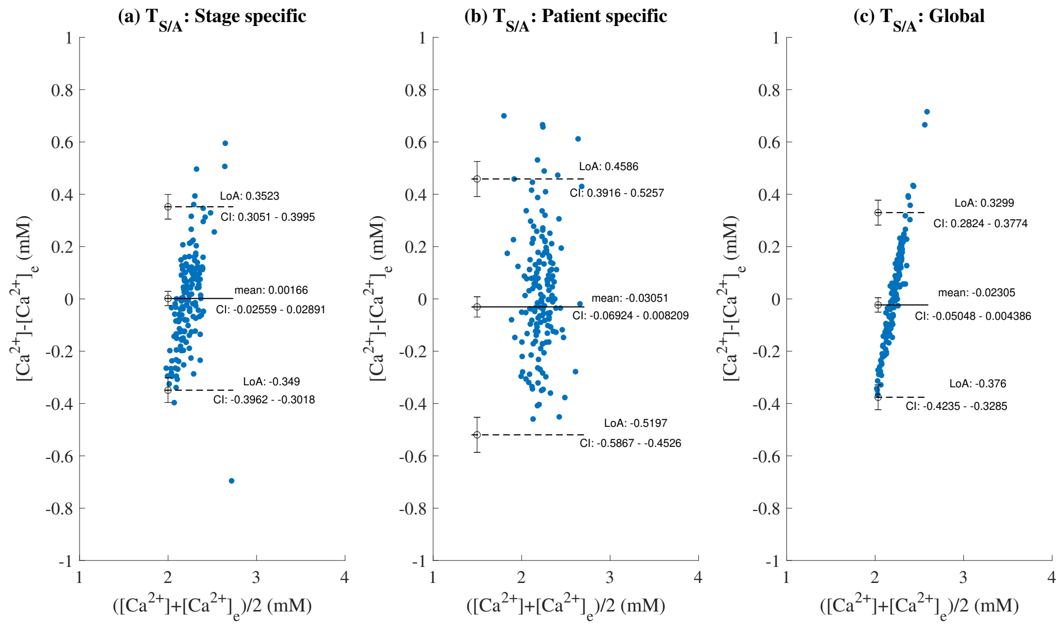

Figure S11. Left Panel: Bland-Altman plot between actual and estimated $[Ca^{2+}]$ for $T_{S/A}$ using stage-specific approach. Middle Panel: Bland-Altman plot using patient-specific approach. Right Panel: Bland-Altman plot using global approach.

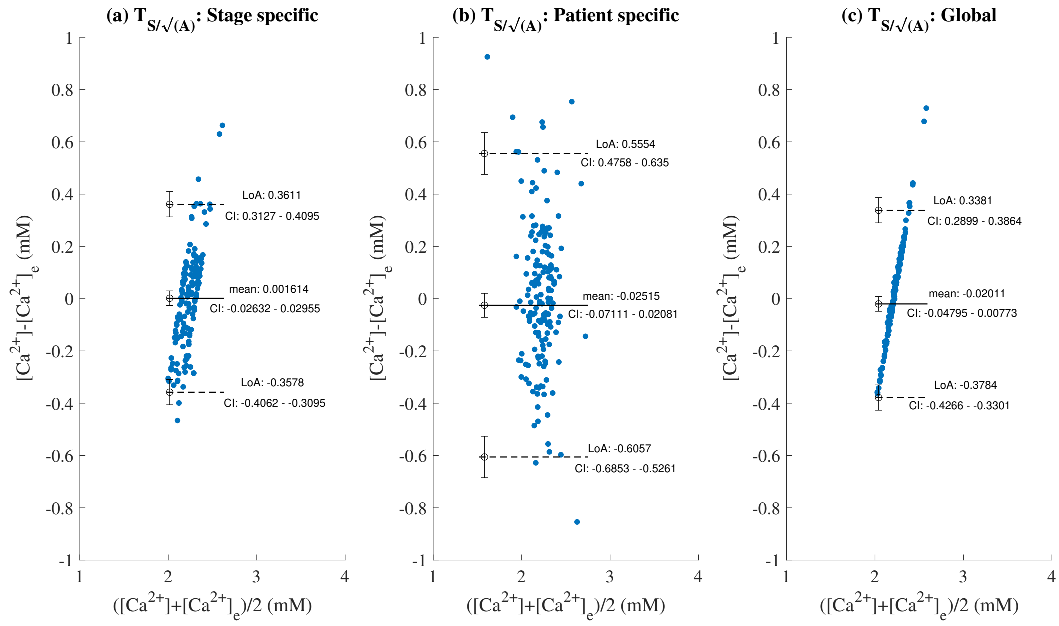

Figure S12. Left Panel: Bland-Altman plot between actual and estimated $[Ca^{2+}]$ for $T_{S/\sqrt{A}}$ using stage-specific approach. Middle Panel: Bland-Altman plot using patient-specific approach. Right Panel: Bland-Altman plot using global approach.

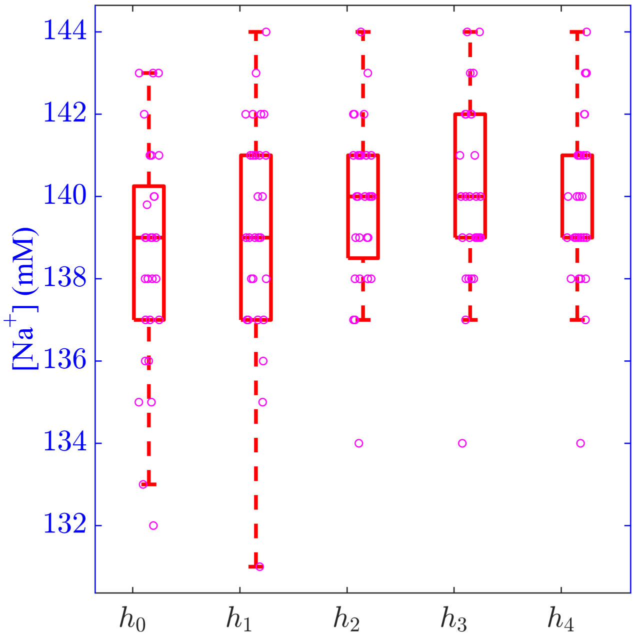

Figure S13. Changes in sodium levels ($[Na^+]$) along HD stages

Table S6. Mean absolute errors (MAE) using stage-specific (S), patient-specific (P) and global (G) approach-based $[K^+]$ estimators, from all patients at all HD time points. Values are expressed as mean absolute error and the units are mM.

MAE	S	P	G
$d^u_{w,Q}$	0.649	0.910	0.821
$d^u_{w,T}$	0.631	0.579	0.763
$T_{S/A}$	0.607	0.770	0.819
$T_{S/\sqrt{A}}$	0.643	0.899	0.895
$d^u_{w,Q}$ & $d^u_{w,T}$	0.539	0.721	0.687

Table S7. Mean absolute errors (MAE) using stage-specific (S), patient-specific (P) and global (G) approach-based $[Ca^{2+}]$ estimators, from all patients at all HD time points. Values are expressed as mean absolute error and the units are mM.

MAE	S	P	G
$d^u_{w,Q}$	0.138	0.127	0.141
$d^u_{w,T}$	0.133	0.098	0.138
$T_{S/A}$	0.135	0.115	0.141
$T_{S/\sqrt{A}}$	0.139	0.130	0.142
$d^u_{w,Q}$ & $d^u_{w,T}$	0.124	0.094	0.139

Table S8. Root mean square errors ($RMSE$) using stage-specific (S), patient-specific (P) and global (G) approach-based $[K^+]$ estimators, from all patients at all HD time points. Values are expressed as root mean square error and the units are mM

$RMSE$	S	P	G
$d^u_{w,Q}$	0.723	1.363	0.961
$d^u_{w,T}$	0.783	0.794	0.946
$T_{S/A}$	0.776	1.108	0.997
$T_{S/\sqrt{A}}$	0.794	1.400	1.053
$d^u_{w,Q}$ & $d^u_{w,T}$	0.771	1.010	0.853

Table S9. Root mean square errors ($RMSE$) using stage-specific (S), patient-specific (P) and global (G) approach-based $[Ca^{2+}]$ estimators, from all patients at all HD time points. Values are expressed as root mean square error and the units are mM.

$RMSE$	S	P	G
$d^u_{w,Q}$	0.173	0.283	0.171
$d^u_{w,T}$	0.164	0.172	0.167
$T_{S/A}$	0.175	0.189	0.178
$T_{S/\sqrt{A}}$	0.180	0.199	0.180
$d^u_{w,Q}$ & $d^u_{w,T}$	0.172	0.116	0.166
